# Applying Infrared Thermography to Soil Surface Temperature Monitoring: Case Study of a High-Resolution 48 h Survey in a Vineyard (Anadia, Portugal)

**DOI:** 10.3390/s20092444

**Published:** 2020-04-25

**Authors:** William Frodella, Giacomo Lazzeri, Sandro Moretti, Jacob Keizer, Frank G. A. Verheijen

**Affiliations:** 1 Department of Earth Sciences, University of Firenze, Via La Pira 4, 50121 Firenze, Italy; giacomo.lazzeri1@stud.unifi.it (G.L.); sandro.moretti@unifi.it (S.M.); 2 Department of Environment and Planning, University of Aveiro, Campus Universitário de Santiago, 3810-193 Aveiro, Portugal; jjkeizer@ua.pt (J.K.); frankverheijen@gmail.com (F.G.A.V.)

**Keywords:** infrared thermography, remote sensing, soil surface temperature, biochar

## Abstract

The soil surface albedo decreases with an increasing biochar application rate as a power decay function, but the net impact of biochar application on soil temperature dynamics remains to be clarified. The objective of this study was to assess the potential of infrared thermography (IRT) sensing by monitoring soil surface temperature (SST) with a high spatiotemporal and thermal resolution in a scalable agricultural application. We monitored soil surface temperature (SST) variations over a 48 h period for three treatments in a vineyard: bare soil (plot S), 100% biochar cover (plot B), and biochar-amended topsoil (plot SB). The SST of all plots was monitored at 30 min intervals with a tripod-mounted IR thermal camera. The soil temperature at 10 cm depth in the S and SB plots was monitored continuously with a 5 min resolution probe. Plot B had greater daily SST variations, reached a higher daily temperature peak relative to the other plots, and showed a faster rate of T increase during the day. However, on both days, the SST of plot B dipped below that of the control treatment (plot S) and biochar-amended soil (plot SB) from about 18:00 onward and throughout the night. The diurnal patterns/variations in the IRT-measured SSTs were closely related to those in the soil temperature at a 10 cm depth, confirming that biochar-amended soils showed lower thermal inertia than the unamended soil. The experiment provided interesting insights into SST variations at a local scale. The case study may be further developed using fully automated SST monitoring protocols at a larger scale for a range of environmental and agricultural applications.

## 1. Introduction 

In earth science applications, land surface temperature (LST) is one of the key physical parameters of land surface processes, from local through to global scales [1,2]. Soil surface temperature (SST) in particular is an important factor that controls the energy exchange processes between the land surface and the atmosphere, representing a fundamental input parameter in meteorological and climatological studies, as well as in hydrological and agricultural analyses [3,4,5,6,7]. SST measures are usually carried out using thermocouples and radiometers [3,7,8]; nevertheless, these devices can present long-term issues regarding logistics, access, and technician costs. In the last few decades, there has been a strong interest in developing methodologies to measure LST and SST using remote sensing (RS) techniques via spaceborne [9,10,11,12,13], airborne [14,15,16,17,18], or ground-based sensors [19,20,21,22]. 

Since the 1980s, thermal sensors from terrestrial, airborne, and spaceborne platforms have been used in a variety of geo-environmental applications [21,22,23,24,25,26,27]. Although the advantages of satellite remote sensing in thermal applications appear to be many, the calibration and validation of the retrieved data is complex and requires complicated correction procedures [28,29,30,31]. To alleviate these issues, ground-based thermal sensors can offer a proper solution for the analysis of the local energy balance and micro-meteorological conditions that can be used as a proxy in large-scale land surface and hydrology experiments in the context of a growing interest for global change research [32,33]. Thermal imaging, or infrared thermography (IRT), offers non-contact, wide-area detection of surface temperatures [34,35,36] and can be used as an alternative or complement to conventional inspection technologies in a wide variety of geo-environmental and agricultural applications [37,38,39,40,41,42,43,44]. Thermal imaging ground-based sensors, such as thermal cameras, have undergone a significant increase in use in recent years, thanks to the technological development of focal plane array uncooled microbolometer sensors, which in turn have allowed for improvement in the accuracy, spatial resolution, and fast measurement of thermal data [45]. 

Soils provide crucial ecosystem services, e.g., supporting human health and habitation, storing carbon, and regulating emissions of greenhouse gases [8]. Climate change and increasing human populations are threatening soil resources and agro-ecological balances, with important implications for food security. Sustainable biochar application to soils [46] has been suggested as a means to mitigate climate change via C sequestration, as well as to improve other ecosystem services, such as crop yields [47,48,49], greenhouse gas fluxes [50,51,52], soil water storage [53,54], soil erosion [55], and soil remediation [56]. However, the effects of biochar application on soil temperature dynamics are not well understood. Yet, soil temperature drives many physicochemical and especially biological soil processes, e.g., soil organic carbon decomposition [57], with climate feedbacks. Therefore, considering the expected changes in global air temperature [58] and precipitation [59], there is an urgent need for a better understanding of both the impacts of increasing soil temperatures in general and the application of biochar to soils as a mitigation and/or adaptation measure. Some authors have shown that soil surface albedo decreases with increasing biochar application rate as a power decay function, with stronger decreases in albedo for dry soil than wet soil [60]. At 50 t/ha, a common biochar application rate, the surface albedo of dry soil decreased by around 30% and that of wet soil by around 18%. All other factors remaining equal, biochar application can be expected to increase SSTs. However, this has not been confirmed by experimental studies so far, with existing studies finding no effect [61], an effect at the surface but not at a 7.5 cm depth [62], or a decrease in soil thermal conductivity but not in soil temperature [63]. Therefore, the impact of biochar application on soil temperature dynamics remains to be clarified [64,65,66]. The lack of available data and their inconsistency is, at least in part, caused by the difficulty in monitoring soil temperature with soil probes in a representative manner given the typically pronounced spatial heterogeneity in key soil variables. The objective of this study was to assess the potential of IRT sensing to overcome this bottleneck, thanks to the scalability of its results, by monitoring SST with high spatial and thermal resolutions in an agricultural application. More specifically, a tripod-mounted IRT camera was used to measure SST with a high temporal accuracy (every 30 min over a 48 h surveying campaign) in a vineyard test site where a biochar field experiment had been set up. Three selected plots with different treatments were analyzed, two of which had sub-surface T probe instruments (5 min measuring interval). A two-spatial-scale approach was adopted: (i) the single-plot scale (given by a 42.5 × 32 cm IRT image); and (ii) the within-plot scale, given by biochar-covered and bare soil spots (from centimetric to millimetric-scale). For this second approach, a semi-automatic procedure in a geographical information system (GIS) environment was adopted for the analysis and the integration of the large IR image dataset. The presented approach can be expanded to the wider agricultural and geo-environmental domain, as well as to larger spatial scales by using IRT sensors based on drone Unmanned Aerial Vehicles platforms. Furthermore, general operative recommendations can be elaborated upon for a fully automated protocol based on an innovative approach using high-resolution spatiotemporal IRT data.

## 2. Materials and Methods

### 2.1. IRT: Theoretical Principles 

IRT (or thermal imaging) is the branch of RS dealing with measuring the thermal radiation of the Earth’s surface features from a distance [45]. Nowadays, most thermal cameras use a focal plane array (FPA) uncooled microbolometer type of detector, mainly because cooling systems make thermal cameras more expensive and less manageable [66]. Microbolometers sensors operate through their response to radiant energy, through changes in their temperature and electrical conductivity, depending on the intensity of absorption of incident radiation [67]. IRT is accomplished by using IR-calibrated cameras (thermal cameras), whose sensors are capable of mapping and monitoring the spatial distribution of thermal radiation [68,69]. An IRT survey produces a matrix of pixel values acquired by the thermal camera array sensor (called a “thermogram” or “thermographic image”), which then undergoes a correction of the sensitive parameters (object emissivity, path length, air temperature, and humidity) by the built-in processor to create a radiant temperature map of the investigated scenario [37,45]. Emissivity can be defined as the ratio of the object’s efficiency at radiating energy compared to a black body (i.e., ε, which is the radiant exitance of an object at a given temperature over the radiant exitance of a black body at the same temperature [70]). While according to Kirchoff’s laws on radiation, black bodies have an emissivity equal to 1, in most remote sensing applications, the objects in question are real bodies (also called grey bodies), e.g., in the 6–14 µm wavelength range, water bodies have very high emissivity values (0.98 < ε < 0.99), whereas soils and rocks have slightly lower values (ε ≥ 0.8) [71]. The concept of emissivity is crucial in IRT because it allows us to relate the IR radiation to an object’s temperature based on Stefan–Bolzmann’s law [45]:

(1) $$W={\varepsilon \sigma T}^{4}$$

where W is the total radiant exitance from an object’s surface, expressed in W·m^2^; σ is the Stefan–Boltzmann constant (5.6697 × 10^−8^ W·m^−2^·K^−4^); and T is the absolute temperature (expressed in K) of the emitting material. Thermal sensors measure the radiant temperatures of objects. The true kinetic temperature of an object (Tkin), generated by the vibration of molecules) can be estimated from its radiant temperature (Trad), provided the object’s emissivity is known [66]:

Trad = ε^1/4^ Tkin (2)

Thermal inertia is a key thermal property that controls the diurnal and seasonal surface temperature variations. It can be thought of as a surface’s ability to conduct and store heat during the day and re-radiate it away at night in the thermal infrared portion of the spectrum [27,66]. Thermal inertia is defined as [72]:

I = (KρC)^1/2^ (3)

where K is the thermal conductivity, ρ is the bulk density, and C is the specific heat capacity of the surface. For rocks and soils, the bulk density (ρ) does not vary significantly, and neither does the specific heat capacity (C) for a dry surface; therefore, variations in thermal inertia are primarily due to variations in thermal conductivity [72]. In remote sensing applications, thermal inertia represents a complex combination of particle size, rock abundance, bedrock outcropping, and the degree of induration [73]; a rough approximation to thermal inertia is sometimes obtained from the amplitude of the diurnal temperature curve (i.e., maximum minus minimum surface temperature) [74]. The thermal inertia has primary effects on the shape of a surface’s diurnal temperature curve: a low thermal inertia surface will experience higher peak temperatures and lower minimum temperatures than a high thermal inertia surface because the low thermal inertia surface becomes heated and cools down faster [75,76]. This is in line with Newton’s law of cooling, which states that the rate of change in the temperature of a body is proportional to the difference between the body’s temperature and the ambient temperature (meaning that the cooling of the body is faster when the temperature difference is higher) [77].

The built-in camera processor and the IRT software (e.g., that from FLIR Systems Inc. [69]) allows for visualizing the thermal pattern image using a false-color temperature scale. The radiant temperature values acquired through an IRT survey do not correspond exactly to the temperatures emitted by the targeted object due to the attenuation and scattering by air molecules, reflection, and emissivity [78,79]. The correction of IRT data requires the calibration of object emissivity values, air temperature and humidity, and the distance between the sensor and the target. In most applications, the emissivity of targeted objects is estimated based on values from the literature; alternatively, there are empirical methods that can be used to estimate emissivity values with a reasonable precision [79,80,81,82,83,84]. 

### 2.2. Study Area and Biochar Treatment Plots 

The study area was a vineyard located within the urban area of the Anadia municipality (Aveiro District, central Portugal) at 40°26′25.91″ N and 8°26′22.90″ W. The climate is warm temperate and mesic [85], with a mean annual precipitation, air temperature, and relative humidity of 700 mm, 15 °C, and ≈ 60%, respectively. At the time of this study (from 1–3 August 2013), the vineyard consisted of mature, five-year-old vines of the Sauvignon Blanc variety under conventional management and without shading. Within the vineyard, an experimental site had been set-up in March 2012 to test the impacts of biochar amendment on key topsoil properties, as well as vine hydric stress. The setup consisted of a total of 12 experimental plots, divided over three blocks, with one control and two biochar application treatments per block. A soil pit was dug in the middle of the experimental site to determine the soil type and to sample the topsoil at a 0–15 cm depth for determining the soil texture (the pipette method), organic matter content (loss-on-ignition method), dry bulk density (using a 5 cm diameter core), and pH (using an electrode in a 1:10 soil:water suspension). For more details, please see Verheijen et al. [55]. The soil was a well-draining Cambisol of 60–80 cm depth with a sandy loam topsoil texture (sand-silt-clay contents of 70%-16%-14%, respectively). The organic matter content of the topsoil was 3.9% (≈2.3% Soil Organic Carbon) [86,87]. The topsoil bulk density was 1.25 g·cm^−3^ and the topsoil pH was 6.0. An HCl test revealed no presence of CaCO_3_, and a molarity of an ethanol droplet (MED) test [88,89] showed the air-dried soil to be wettable. The biochar that was applied in the experimental site was produced from mixed wood sievings in a Pyreg^®^ 500 III pyrolysis unit (Dörth, Germany), 620 °C maximum temperature, 20 min duration, and a H/C ratio of 0.18 (Swiss-Biochar GmbH, Basel, Switzerland). The median particle size was 29.5 µm with the following particle size distribution (*w*/*w*): 4% (<0.1 mm), 25% (0.1–0.5 mm), 34% (0.5–2 mm), 37% (>2 mm). The pH was 10.4 and the ash content was 28% (full characterization in Prodana et al. [90]).

### 2.3. Experimental Setup

**Figure 1:**
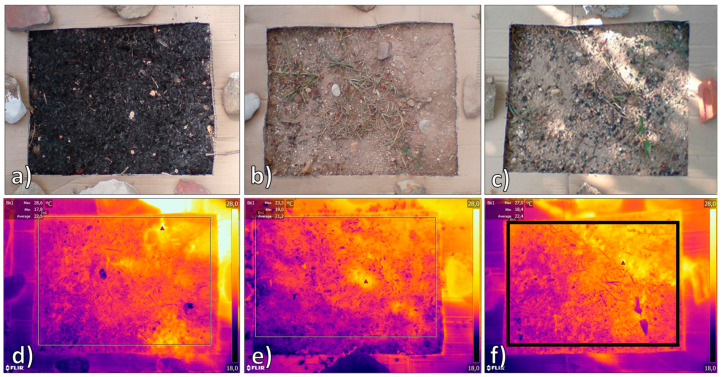
Examples of the analyzed plots: plot B (**a**), plot S (**b**), and plot SB (**c**). Corresponding infrared thermography (IRT) images underneath (**d**–**f**). The black square in panel f shows the surveyed IRT sector (region of interest (ROI)).

**Figure 2:**
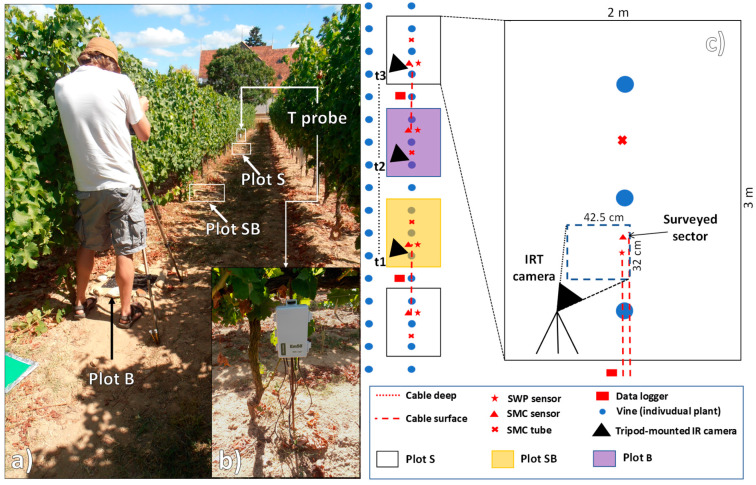
Experimental setup in the vineyard study area: (**a**) tripod-mounted thermal camera, (**b**) Decagon Em50 data logger, and (**c**) vineyard control treatment soil plot.

The rationale behind the present study was to assess the effect of a biochar amendment on the vineyard SST and to relate the SST patterns to simultaneous records of air temperature and relative humidity data from a standard weather station, as well as with sub-surface soil temperature data from automatic sensor probes. For this study, the three plots of one of the vineyard blocks were selected (Figure 1). These plots were characterized by different treatments: the control plot was dominated by bare soil (namely plot “S”); one plot had biochar (a soil amendment) applied to the surface, providing a 100% soil cover (namely plot “B”); while the third plot had biochar incorporated into the topsoil, providing a soil cover of 10% (namely plot “SB”). The original biochar application rates were 40 and 4 t·ha^−1^ for plots B and SB, respectively. A tripod-mounted IRT camera was used to measure the SST with a high temporal accuracy (30 min) on all three plots over a 48 h surveying campaign, starting with plot B and ending with plot SB, from 08:00 on 1 August 2013 to 09:00 on 3 August 2013. The instrument employed in this work was a tripod-mounted SC620 thermal camera (FLIR Systems, Wilsonville, OR, USA) [68]. This device can measure electromagnetic radiation in the Long Wawelenght InfraRed band, with a 640 × 480 pixel detector size and a spatial resolution of 0.65 mrad (see Table 1 for further specifications). The IR camera was mounted on a tripod at a 1.3 m height above the soil surface, leading to an image geometrical resolution of 0.85 mm (Figure 1 and Figure 2). Sunrise and sunset during this period were at 06:24 and 20:41, respectively. Furthermore, plots S and SB were had two T-probe instruments at a 10 cm depth and were ≈ 15 cm apart, including (i) a Degacon 5TE probe (Pullman, WA, USA), which combines soil moisture content *v/v* (capacitance sensor) with electrical conductivity and temperature (0.1 °C resolution), and (ii) a Degacon MPS2 probe, which combines soil water potential (dielectric water potential) with temperature (0.1 °C resolution). The readings of the probes were recorded at 5 min intervals using Decagon EM50 loggers (Figure 2). In this study, the mean values of the two probes (ST10) per plot were used. Local weather data (air temperature, relative humidity, rainfall) were acquired using a pocket thermohygrometer (OM-EL-USB-2-LCD datalogger; Omega Engineering, Manchester, UK) located at a 1 m height above the soil and acquiring with 5 min intervals (Figure 2), and using the Instituto Português do Mar e da Atmosfera (IPMA) Anadia weather station [91] located approximately 190 m away from the test site (Table 2). 

**Figure 3:**
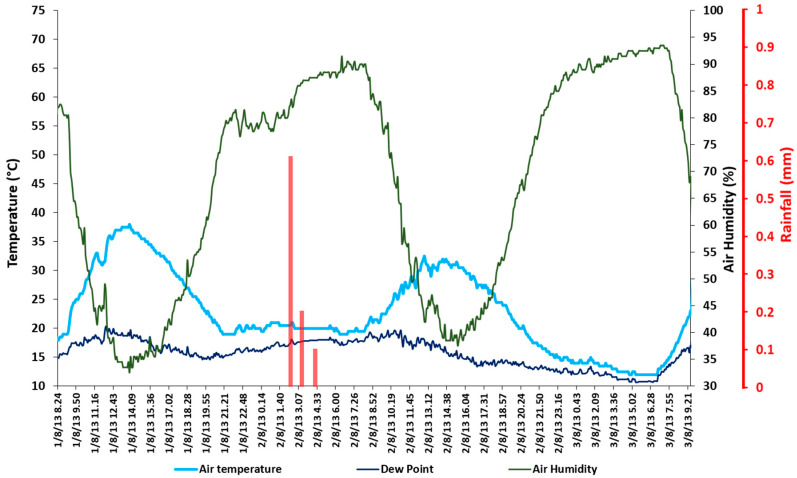
Pocket thermohygrometer weather data and hourly precipitation from the IPMA station.

The acquired SST dataset was compared to the soil sub-surface temperature data set from the soil probes, as well as to local weather conditions. Based on these weather data, the monitoring period was divided into two sub-periods: a first one from 08:00 on 1 August to 02:00 on 2 August, which was characterized by dry atmospheric conditions and clear sky, and a second one from 02:00 on 2 August to 09:00 on 3 August, which started with a low-intensity precipitation event that lasted approximately 2 h and produced 0.9 mm of rainfall (Figure 3).

Emissivity values of the topsoil and biochar were retrieved using laboratory tests following the “reference emissivity material method,” as given in References [82,83,84]. The method briefly consists of attaching a reference emitter to the samples and heating both to 20 °C above room temperature; the unknown emissivity of the samples is then measured through the IRT software using a calibration procedure based on the known reference value. The obtained results (ε = 0.96 for the soil and ε = 0.98 for the biochar) agreed well with the values in the existing literature [79,80,92,93,94,95]. These emissivity values were then used to correct the plot thermograms using the FLIR Tools + software [69]. The latter was also used for the single-plot scale analysis to measure the maximum, minimum, and mean SST for the acquired IRT dataset by using the square tool function on the plot area of the thermograms (Figure 1f). 

**Figure 4:**
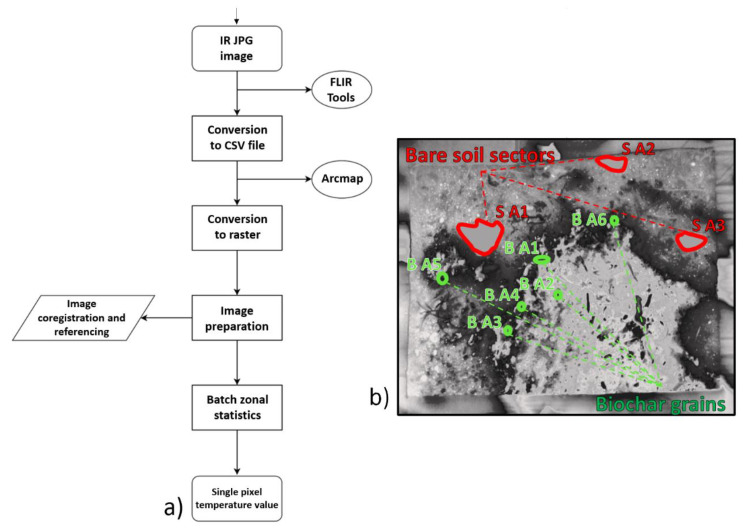
Methodological workflow (**a**) and analyzed ROIs on the plot SB thermogram (**b**): bare soil sectors (red polygons) and biochar clusters (green polygons).

To analyze the effect of biochar grains on the SST of the SB plots, a “within-plot scale” analysis was performed on the whole IRT dataset using a semi-automatic procedure in a GIS environment. Regions of interest (ROIs) from B1 to B6 for biochar and from S1 to S3 for soil were manually selected on the SB thermogram. The criterion used to establish these areas was determined by the necessity of having SST only representing the investigated material. Therefore, biochar clusters were chosen to be represented by a coalescence of three or more grains. Similarly, the bare soil sectors were selected in a way that avoided the presence of rock clasts grains/pebbles or vegetation. These criteria were used to avoid the thermal influence caused by different materials (e.g., the cooling effect of vegetation due to evapotranspiration). To analyze the SST in detail, the time series were analyzed on three selected biochar-clustered grains and bare soil sectors each within the SB plot, and the thermograms were manually converted to a raster format, spatially co-registered, and analyzed using the ESRI ArcGIS software “zonal statistics tool” [96] (Figure 4). The tool was run in batch mode to automatically extract the SST values from the plot’s entire thermogram dataset, both for the thermograms and the single polygon representing the ROIs. For the pixels located in the same area, the tool computes the mean temperature, reports the minimum and maximum temperatures, and their range. As these values are extracted from all the thermograms, they were recorded in a table for each polygon separately to allow for the monitoring of SST data for the specific ROI.

## 3. Results 

### 3.1. SSTs from IRTs

**Figure 5:**
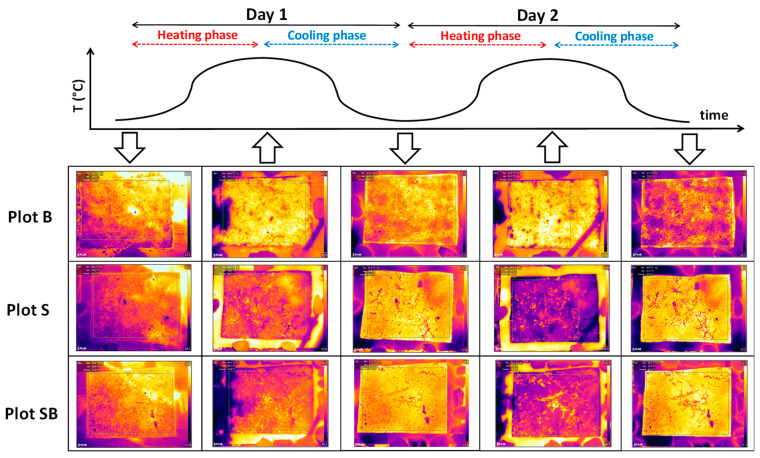
IRT dataset of the analyzed plots showing the SST variations on the single thermograms.

**Figure 6:**
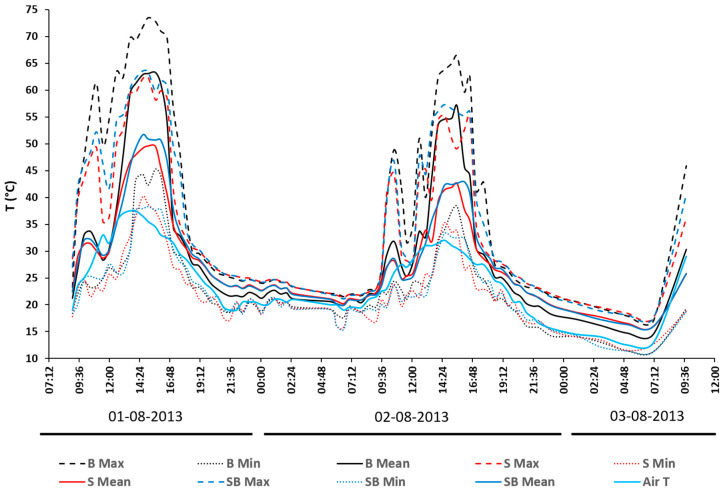
Maximum (dashed lines), minimum (dotted lines), and mean (continuous lines) soil surface temperatures monitored during the survey. Plot B (100% biochar cover)—black lines, plot S (bare soil)—red lines, plot SB (mixed soil-biochar)—dark blue lines, and air—light blue line.

The analyzed IRT dataset showed a double-humped daily SST distribution, characterized by four heating and cooling phases during the 48 h monitoring period (Figure 5 and Figure 6). The air temperature (air T) peaked around 14:00 on day 1 (36.5 °C), while the maximum SSTs for all the plots peaked about an hour later, i.e., at 15:04 (73.5 °C for plot B, 62.4 °C for plot S, and 63.4 °C for Plot SB). After these peaks, the SSTs dropped quickly for all the plots from around 16:00 to 19:00 (≈ 30 °C), when the differences with air T were smallest (this was because direct sunlight stopped hitting the soil surface from 17:00). The SST peaks continued to drop during the first night down to 19 °C at 06:30 on day 2, followed by a sharp increase when direct sunlight started reaching the plots again, starting from 08:30. During day 2, the effects of rainfall from 02:30 to 04:30, as well as the increased air humidity (Figure 3) and cloud cover, were clearly visible in the cooling of all the treatment plots. 

The air T peaked at 12:54 (32.5 °C) and the peak SSTs of the treatments were staggered, i.e., plots S and SB at 14:34 (55.4 and 57.3 °C, respectively) and plot B an hour later at 15:34 (66.3 °C). Moderate and short T drops were visible in both daily cycles from 10:30 to around 12:30, resulting from the vine canopy shading. These drops were more pronounced in the maximum SSTs than in the minimum SSTs (T min) and were the least marked in the mean values (T mean). On day 2, this drop lasted longer than on day 1, i.e., from 11:30 to 13:00 as opposed to from 12:30 to 13:30. This was probably due to a cooling effect from the early morning rainfall.

During the nighttime of day 2, the difference between the air T and SST increased again from 19:00 to 05:00, while the rain during the first night quickly equalizes the air and SSTs (from 03:00 to 08:30). By comparing the shape of the distribution for the air T and SST, it can be seen how the former had a more regular pattern of heating and cooling, and how the variations in sun heating required a specific time to be observed on the distribution. Compared with the air T, the latter was evidently sharper and less homogeneous, presenting steep sides and peak values when related to the distribution of the air T. The minimum values of both the air T and SST occurred between 06:30 and 07:00 on both surveying days (day 1: 17.9 °C for the air T and 21.5, 21.4, and 21.1 °C for plots S, B, and SB, respectively; day 2: 13 °C for the air T and 17.1, 17.7, and 17.6 °C for plots S, B, and SB, respectively). This coincided with the time just after sunrise and before sunlight hit the plots again. The only time when the SST was lower than the air T was from 11:30 to 12:00, i.e., during the time when the vine canopy shaded the soil.

### 3.2. Soil Temperatures at a 10 cm Depth

**Figure 7:**
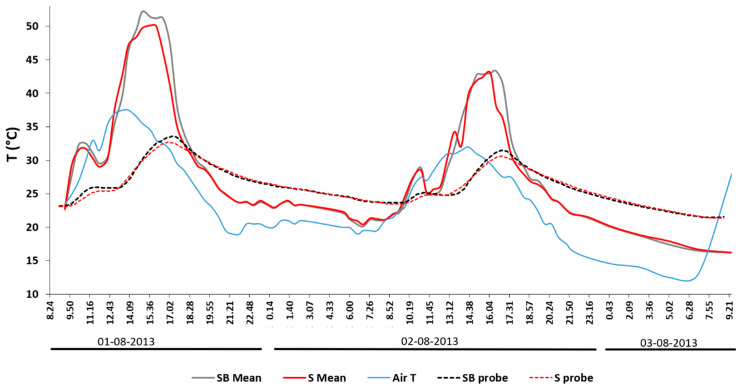
Comparison between the air temperature, kinetic temperature at a 10 cm depth (S and SB probes), and mean kinetic soil surface temperature of biochar clusters and soil sectors within plot SB, obtained by converting the Trad measured by the IRT camera into Tkin.

**Figure 8:**
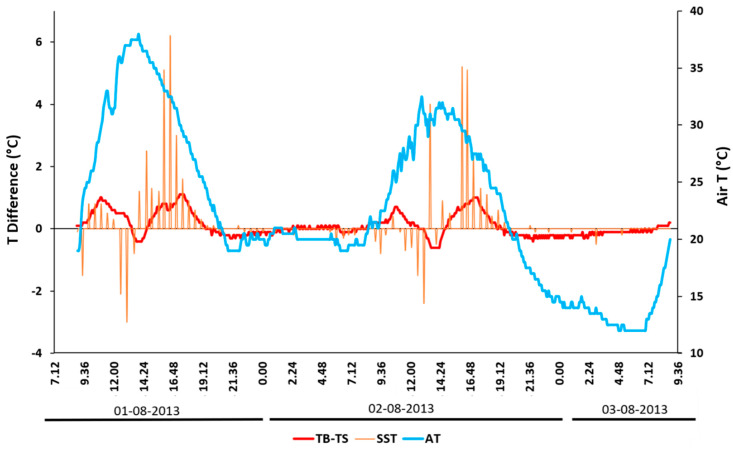
Biochar effect on soil temperature, obtained by subtracting the bare soil (S) temperature from the biochar-amended soil (SB) temperature (TB − TS, read on primary *y*-axis), for both soil surface (orange) and a 10 cm depth (red), with air temperature (air T) in blue (read on secondary *y*-axis).

At a 10 cm depth, the SB plot reached a T max that was 0.9 °C higher than the S plot around 18.00 on day 1 and 19.00 for day 2 (3 h after the air T and 1.5 h after the soil surface T; Figure 7). This indicates that plot SB warmed up and cooled down more quickly. From its peak, the T of plot SB seemed to decrease more quickly, leading to a slightly lower T from 20:00 onward. The stronger cooling seemed even more obvious for the canopy shading time in the morning, particularly on day 2. Additionally, by comparing the elapsed time between the SST and the air T, on day 2, the time interval was greater, and the peaks had wider bases compared to day 1. Figure 8 shows the differences in SST, as well as the soil temperature at a 10 cm soil depth between the control plot (S probe) and the plot with biochar mixed into the topsoil at an agronomically relevant 40 t·ha^−1^ application rate (SB probe). Positive values indicate a heating effect, with negative values indicating a cooling effect. 

It can be observed that the temperature changes at a 10 cm soil depth occurred with a slight lag to SST and were several times smaller in magnitude. The air temperature did not appear to drive biochar effects on soil temperatures.

### 3.3. SST Raster Analysis for a Spot in Plot SB

**Figure 9:**
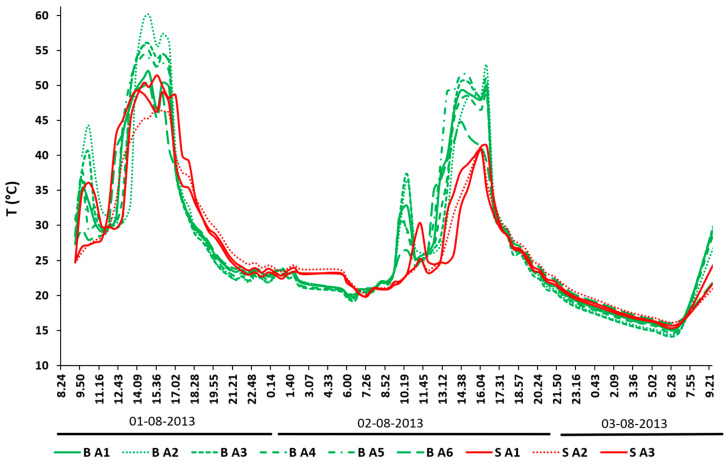
SST comparison between the ROIs in plot SB: green lines represent biochar grains (B), while red lines represent bare soil sectors (S).

The SST data from the six biochar (grains/clusters) and three bare soil spots in the SB plot are shown in Figure 9. The biochar areas (B label in Figure 9) show a similar thermal pattern, as well as the bare soil sectors (S label in Figure 8). The different thermal inertias of the materials (lower in the biochar, higher in the soil) were clearly visible in the daily peaks of the SST pattern (around 13 °C of variation in the 13:00–15:00 interval) and at night time (around 5 °C in the 01:30–05:00 interval), when the lowest temperatures were measured.

**Figure 10:**
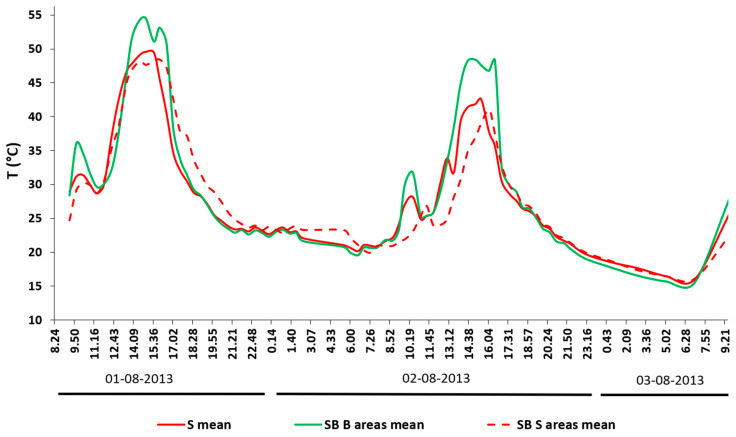
Mean SST variations between the bare soil in plot S (red), biochar cluster/grains in plot SB (green), and bare soil sectors in plot SB (dashed red).

**Figure 11:**
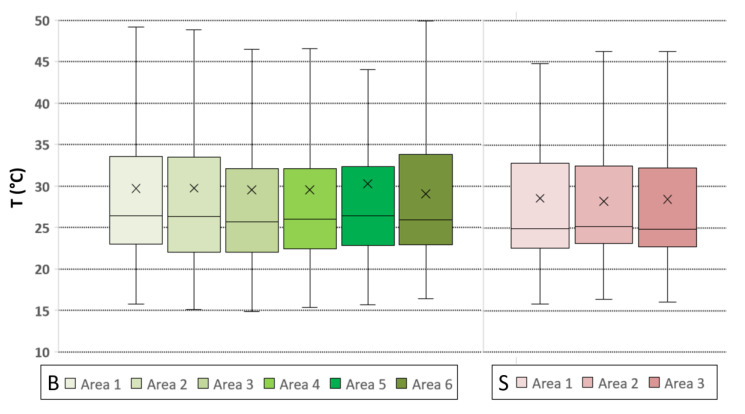
Boxplots for the biochar (in green tones) and bare soil areas (in red tones) in plot SB showing the mean temperature distribution.

This was confirmed by analyzing the mean SST variations of biochar grains/clusters and bare soil sectors (Figure 10), where biochar showed stronger variations in the heating and cooling cycles than bare soil; the biochar’s lower thermal inertia was due to the material’s coarser texture and grain size, influencing the pore size distribution, and therefore, the air circulation. It can be seen how its temperature rose more quickly around the zenith, i.e., during the strongest solar radiation hours. Similarly, the temperature dropped after the solar irradiation decreased because of its lower thermal capacity compared to the soil. To provide a descriptive distribution of the temperatures for plot SB, box plots representing each ROI in the plot are also reported (Figure 11). Generally, both plot types had a denser distribution of the values in the lower portion, as highlighted by the median, showing the non-normal distribution of temperatures (as shown by the mean represented using the symbol “×” in the boxes). Biochar boxplots showed higher maximums and generally a wider range of minimum values compared to the soil boxplots, as well as a greater interquartile range, testifying a general tendency toward extreme temperatures, which is also evidenced by the error bars (that depict a difference between soil and biochar areas of up to 10 °C).

## 4. Discussion 

Analyzing the SST daily variations can have important implications for agronomy and environmental studies since the soil temperature drives soil physicochemical and biological processes. Traditionally, soil temperature data is gathered using soil probes at varying soil depths; however, the spatial heterogeneity of key soil properties at various scales hinders representative soil monitoring using point data. IRT can potentially overcome this limitation thanks to its scalability, and thereby improve our understanding of land and soil management via understanding the soil temperature. 

IRT was used similarly by previous authors, but rather than to assess the SST, it was used in laboratory experiments to estimate the soil surface microrelief and rill morphology [97], to map soil surface permeability and identify preferential flow [98], to map macroporosity at the soil surface [99], to assess cooling the soil surface with cold water for assessing soil water repellency [100]. This paper proposed a novel, scalable approach for applying IRT monitoring of SST in environmental applications. In particular, the SST patterns of selected plots (biochar plot B), bare soil (plot S), and biochar-amended soil (plot SB) of a vineyard test site in a warm temperate environmental zone (Anadia, Portugal) were analyzed over a 48 h period. This was characterized by dry (day 1) and wet conditions (day 2) (Figure 5), which were due to a minor rainfall event that occurred during 2:00–4:00 on day 2 (Figure 3). The IRT data were acquired at high spatial and thermal resolutions (sub-millimeter and hundredth of a degree Celsius, respectively) with 30 min intervals using a tripod-mounted infrared thermal camera equipped with a focal plane array uncooled microbolometer. The purpose was to test the effect of biochar on the soil thermal properties since biochar can be applied to soils to retain more water and to improve the quality and fertility of the soil [47,48,49,50,51,52,53,54,55,56,101]. Due to its cost-effectiveness, portability, and easy battery power supply, this type of sensor was more suitable for this specific application as a cooled system.

**Figure 12:**
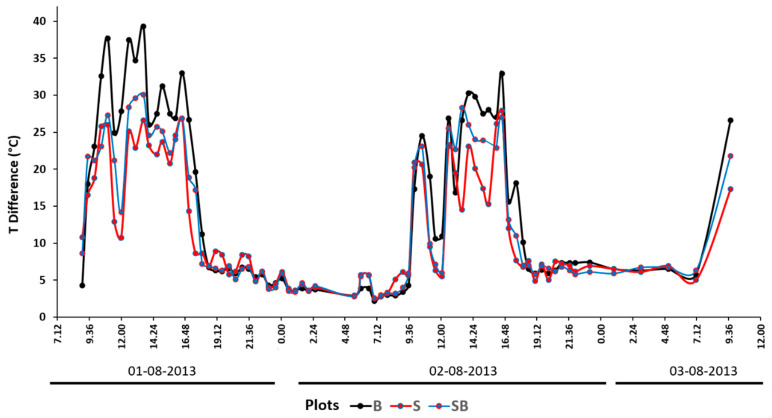
Temperature range within the IRT images for plots B (black), S (red), and SB (blue).

The results of the experiments showed that with the 100% biochar cover (plot B), the SST reached a higher daily temperature peak relative to the other plots, and showed a faster rate of T increase during the day (especially in the last part of the rise toward the peak). This effect may be explained by the reduction in soil surface albedo, which according to the dose–response study of Verheijen et al. [61], was expected to be in the range of 80%. However, on both days, the SST of plot B dipped below the T of the control treatment (plot S) and the biochar-amended soil (plot SB) from about 1800 onward and throughout the night. This thermal behavior was likely connected to the biochar’s higher emissivity and a lower thermal inertia due to coarser and loose grains that allowed for a lower resistance to air circulation relative to soil [47,97,98]. This thermal behavior was further supported by analyzing the range in the SST graph for all three plots (Figure 12). Plot B had a larger range compared to plots S and SB, which was caused by the higher thermal inertia of the soil in both plots. This is also supported by the dose–response study of Verheijen et al. [61], which showed that the biochar concentration in plot SB was expected to result in a soil surface albedo reduction of 30%–40%. This effect on SST variation was also evident when biochar was mixed with soil (plot SB), even at a 10 cm depth (Figure 7 and Figure 8), as noted by the fast response of the T probe. The interpretation of the subsurface soil temperature data was limited by the probe’s resolution (i.e., 0.1 °C) and potential minor differences in the installation depth. The latter was compensated for to some extent by using the mean of two side-by-side probes for each plot. The effect size of the higher T peaks was several times the size of the probe resolution, which strengthened our confidence in the results. The temporal resolution of this case study is also relevant to experiments in soil science aimed at determining whether, and by how much, biochar application may increase the soil T, which is an important driver of soil biological processes, including relevant implications for soil respiration [102] and evaporation [103] at the effect size on soil T that we found here. At the same time, regarding the interpretation of the soil SSTs, the adopted thermal camera thermal sensitivity was 0.04 °C, while the observed effects on the measured values were many times larger. The confidence in this would also increase through upscaling in future studies. A drawback of this exploratory study is that we did not have physical replicates; therefore, new experiments in a similar climatic context will be planned to enhance confidence in the interpretation of the SST effects, also through upscaling to a larger scale of observation. Further investigations with more and higher-resolution T probes are recommended to further our understanding of how the SST regimes relate to temperature regimes within the soil profile, and in particular, how biochar impacts this relationship. The effect of rainfall on the SST of all the graphs was particularly evident in day 2 when the soil water content increased, and therefore, all of the temperature peaks decreased, the diurnal temperature fluctuation range was reduced, and the SST patterns tended to homogenize following the second peak. 

The “within-plot scale” analysis in plot SB allowed for assessing the thermal behavior of single picture elements over the entire monitoring period, and consequently, getting a better understanding of the thermal differences between bare and biochar-covered spots of the soil surface. 

The approach used to extract the spatial information from the IRTs showed sharp peaks in the cooling and heating cycles for biochar, confirming its different thermal behavior relative to bare soil, and suggesting that biochar grains can modify the thermal properties of the topsoil by lowering its thermal inertia relative to bare soil. Indeed, the thermal behavior of the single bare soil sectors in the Plot SB showed the same thermal behavior (SST heating/cooling phases evolution and maximum and minimum values) as plot S. The same thermal behavior was not found for the biochar grains because individual biochar grains had the same thermal behavior but a different SST range compared to the whole B plot. 

## 5. Conclusions

The adopted methodology has shown how its high temporal and thermal resolutions can provide useful insights for the understanding of daily microclimate variations in a warm temperate environmental zone; it also allowed for observing differences related to one specific type of soil management practice. The abilities regarding data collection, as well as management and soil properties determination, teamed with agronomists’ knowledge of how the influence of such parameters on vineyards, allows for developing special microculture methods to increase the productivity of plantations. IRT has shown the potential to upscale soil T data to the crop scale and as input for precision farming operations. For biochar field experiments, it may be especially useful to improve the spatial resolution of soil T data and support furthering our understanding of biochar’s effects on soil T dynamics. Although the proposed methodology was applied to soil plots, in future research, the raster approach allows for an upscaling of the procedure through the application of the sensors on a drone platform. This, combined with the high resolution of the infrared sensor. will allow for extending the analysis of larger areas, such as the whole vineyard/plantation. To achieve such a goal, a proper number of thermohygrometer sensors should be placed in the investigated area to collect the necessary parameters for the thermogram image corrections. This procedure will also require the placing of georeferenced optical markers that are necessary for the composition of the acquired images mosaic. The SST analysis will require the creation of mask features to analyze only the soil areas by excluding the vegetated portion; this could be achieved through the application of a supervised classification of the area in a GIS environment. Through the acquisition of multiple images with a designated time step and thanks to the batch analysis, it can be possible to obtain a time-dependent variation of the SST (theoretically, by computing a raster difference, we could obtain a map showing the highest soil T for the analyzed time). Future developments of the procedure can lead to a fully automated protocol that can monitor the interested areas on a daily basis. 

## Figures and Tables

**Table 1:** FLIR SC620 thermal camera main technical specifications.

Feature	Unit	Value
Detector size	pixel	640 × 480
Spectral range	µm	(7.5, 13)
Temperature range	°C	(−40, +500)
Thermal accuracy	°C	±2
Thermal sensitivity	mK	40
Field of view (FOV)	°	24 × 18
Lens	°	24
Spatial resolution	mrad	0.65
Minimum focus distance	m	0.3
Image frequency	Hz	30

**Table 2:** Weather data from the Instituto Português do Mar e da Atmosfera (IPMA) Anadia station. T: Temperature, RH: Relative humidity.

Heading	1 August	2 August	3 August
T med (°C)	22.0	21.4	19.1
T max (°C)	31.9	28.5	26.6
T min (°C)	14.9	14.6	12.1
RH med (%)	75	75	75
RH max (%)	98	100	99
RH min (%)	36	39	45
Daily Rainfall (mm)	0.0	0.9	0.0
